# Mesenchymal stem cell therapy targeting mitochondrial dysfunction in acute kidney injury

**DOI:** 10.1186/s12967-019-1893-4

**Published:** 2019-05-02

**Authors:** Lingfei Zhao, Chenxia Hu, Ping Zhang, Hua Jiang, Jianghua Chen

**Affiliations:** 1 Kidney Disease Center, The First Affiliated Hospital, College of Medicine, Zhejiang University, Hangzhou, Zhejiang People’s Republic of China; 2 Key Laboratory of Kidney Disease Prevention and Control Technology, Hangzhou, Zhejiang People’s Republic of China; 3 Institute of Nephrology, Zhejiang University, Hangzhou, Zhejiang People’s Republic of China; 4 State Key Laboratory for Diagnosis and Treatment of Infectious Diseases, The First Affiliated Hospital, College of Medicine, Zhejiang University, Hangzhou, Zhejiang People’s Republic of China

**Keywords:** Mesenchymal stem cells, Mitochondrial dysfunction, Acute kidney injury

## Abstract

Mitochondria take part in a network of cellular processes that regulate cell homeostasis. Defects in mitochondrial function are key pathophysiological changes during acute kidney injury (AKI). Mesenchymal stem cells (MSCs) have shown promising regenerative effects in experimental AKI models, but the specific mechanism is still unclear. Some studies have demonstrated that MSCs are able to target mitochondrial dysfunction during AKI. In this review, we summarize these articles, providing an integral and updated view of MSC therapy targeting mitochondrial dysfunction during AKI, which is aimed at promoting the therapeutic effect of MSCs in AKI patients.

## Background

Acute kidney injury (AKI) is a common clinical disorder that is defined as a rapid renal function decline accompanied by the dysregulation of extracellular volume, electrolytes and possibly multi-organ failure [1]. Multiple initiating insults may induce AKI, including ischemia/reperfusion (I/R) injury, drug administration, cardiovascular surgery and sepsis. For numerous reasons, the prevalence of AKI is as high as 5 to 7% in hospitalized patients and is over 30% in ICU patients [2, 3]. In addition to the high morbidity rate, current therapy for AKI is still confined to preventive strategies and supportive care, such as dialysis and renal transplantation [4]. All these factors make AKI a costly disease, placing a heavy burden on the public health care system [5].

Recent evidence highlights mitochondrial dysfunction as playing an important role not only in mitochondrial diseases but also in the pathogenesis of many other diseases, such as neurodegenerative disorders, cardiac diseases, sepsis, cancer and diabetes [6–9]. The kidneys, especially the proximal tubules and medullary thick ascending limbs, largely rely on oxidative metabolism to provide the vast amount of adenosine triphosphate (ATP) they require for tubular reabsorption [10]. Therefore, it is not surprising that mitochondrial dysfunction is central to the pathogenesis of AKI, regardless of whether I/R injury, sepsis or exposure to toxic reagents is the initiating insult [11, 12].

Mesenchymal stem cells (MSCs) are fibroblast-like cells, which are characterized by their robust self-renewal, regenerative, proliferative, and multilineage differentiation abilities [13]. During the last decade, MSCs have emerged as potential candidates for tissue repair. Several preclinical studies have demonstrated the benefit of MSC therapy in AKI models. The main mechanism underlying the beneficial effects of MSCs in AKI is their paracrine/endocrine activity, which may exert anti-apoptotic, immunomodulatory, anti-oxidative and pro-angiogenic effects [14–17]. Recently, accumulating evidence has emphasized that the restoration of mitochondrial function can also be a potential therapeutic target for MSCs intervention in tissue repair [18–20]. It is unknown whether MSCs exert similar effects in AKI. In this review, we summarized the most recent data regarding the role of mitochondrial dysfunction in the development of AKI and discussed the possibility of MSCs relieving mitochondrial dysfunction for the treatment of AKI.

### Mitochondria and their dysfunction in AKI

Mitochondria are fundamental organelles that are present in almost all eukaryotes. Mitochondria have a unique structure characterized by a highly permeable outer membrane and a highly impermeable inner membrane. The basic function of mitochondria is the generation of ATP through oxidative phosphorylation (OXPHOS), but an increasing number of studies have demonstrated that mitochondria also play essential roles in cell proliferation, the modulation of intracellular reactive oxygen species (ROS), calcium homeostasis and apoptosis [21]. The density and distribution of mitochondria vary in different tissues because they are determined by the different levels of energy demand. Due to its physiological function of blood purification, the kidney is an organ with abundant mitochondria, consuming approximately 7% of the daily ATP expenditure in the body [22, 23]. Its high metabolic requirement makes the kidney an energy-sensitive organ, and mitochondrial dysfunction is very common during AKI, especially in the proximal tubules, which lack the capacity to perform anaerobic glycolysis [24].

**Fig. 1:**
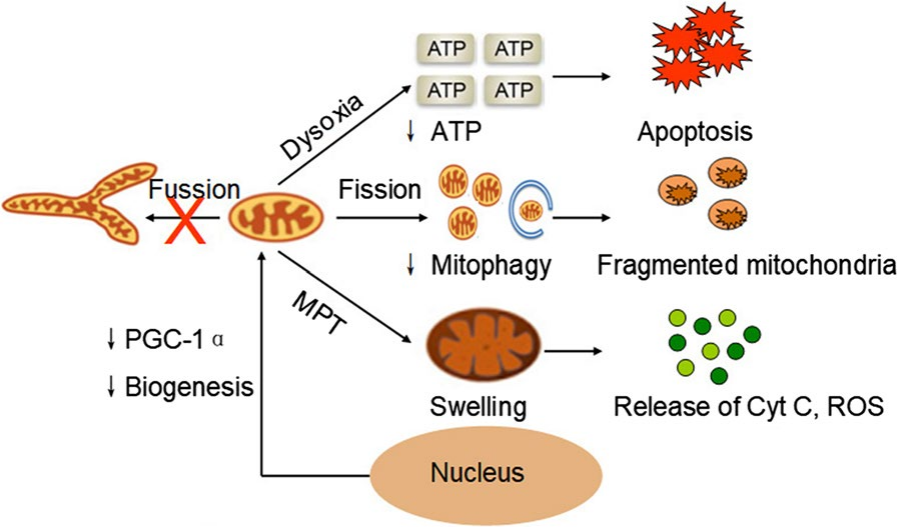
Mitochondrial dysfunction during AKI. During AKI, the mitochondria tend towards fission rather than fusion. Together with the suppression of mitophagy, fragmented mitochondria are observed in the cytoplasm. Downregulation of PGC-1α inhibits the biogenesis of mitochondria. Mitochondrial swelling is regarded as a consequence of MPT, which may subsequently release Cyt C and ROS. Dysoxia is also very common, inducing a reduced generation of ATP and cell apoptosis. *PGC*-*1α* peroxisome proliferator-activated receptor-γ coactivator-1α, *MPT* mitochondrial permeability transition, *ATP* adenosine triphosphate, *Cyt C* cytochrome C, *ROS* reactive oxygen species

Whether mitochondrial dysfunction is an initiator of AKI or a contributor to the process is very difficult to determine. However, it seems possible that related risk factors first induce mitochondrial injury, which may then contribute as an additional mechanism in the pathophysiological process of AKI. I/R-induced AKI is an example of this. During the ischemic phase, due to the limited blood and oxygen supply, OXPHOS is hampered. The production of succinate in the mitochondria is increased as a compensatory mechanism for the reduction of NADH and coenzyme Q. Once the blood supply has recovered, the accumulated succinate is reoxidized, and ROS are rapidly generated in the mitochondria through reverse electron transport along the electron transport chain [25]. The burst of ROS generation in the mitochondria is widely recognized as a dominant injurious effector, which may then activate a plethora of signaling pathways in cells, causing the histologically characteristic inflammation and necrosis [26, 27]. All this damage is in accordance with injury to a wide range of tissues, including renal tissues, finally resulting in renal failure [28]. In the following section, we will discuss recently updated findings about mitochondrial dysfunction during AKI and the underlying mechanisms, including histological changes, mitochondrial biogenesis, dynamics and mitophagy (Fig. 1).

#### Histological changes

Mitochondria-centered ultrastructural changes, together with metabolic and bioenergetic alterations, play central roles in the development of AKI. It is worth noting that some changes even appear to precede the clinical manifestations of AKI. Renal pathological examinations of patients who died from shock, trauma and sepsis revealed swollen mitochondria as well as autophagosomes in affected tubular cells [29, 30]. Similar results were also found in renal tissue from patients undergoing controlled renal ischemia, such as partial nephrectomy [31]. Mitochondrial swelling is regarded as a consequence of mitochondrial permeability transition (MPT), which is activated by Ca^2+^ overload and oxidative stress. Swollen mitochondria may subsequently release mitochondrial intermembrane proteins, triggering the next steps in cell death [32]. Apart from mitochondrial swelling, a decrease in mitochondrial abundance in proximal tubular cells was observed after exposure to cisplatin or I/R, which may result from mitochondrial fragmentation [12].

Ultrastructural changes in mitochondria are accompanied by metabolic and bioenergetic dysfunction. For example, treatment with cisplatin might induce the release of cytochrome C (Cyt C) in proximal tubular cells [12]. The widespread loss of mitochondrial respiratory proteins and electron transport chain enzymes was also observed in multiple animal AKI models [11, 33]. Dysoxia, which is dysfunction of the mitochondrial utilization of oxygen, was not only present in sepsis but also in postoperative AKI, as determined by Ricksten et al. [34]. All of these factors hinder the utilization of fatty acids, which are the main energy source for OXPHOS in the renal cortex, inducing fat accumulation in the proximal tubules and reduced ATP production [35].

Mitochondria are the major intracellular source of ROS. During normal OXPHOS, the content of converted superoxide radicals is usually < 4% [36]. However, excess production of ROS by the mitochondria has been observed during tubular injury in AKI [37]. Based on this, mitochondria-targeted antioxidants, such as mito Q and Szeto-Schiller (SS) peptides, have been shown to have a promising renoprotective effect in AKI in recent years [38, 39].

In conclusion, multiple pieces of evidence have suggested the existence of ultrastructural, metabolic and bioenergetic changes in mitochondria during AKI, and sustaining mitochondrial homeostasis is the basis for maintaining stable function [40]. In the following section, we will discuss the role of mitochondrial biogenesis in AKI.

#### Mitochondrial biogenesis

Mitochondrial biogenesis is an important process for maintaining mitochondrial homeostasis. Through mitochondrial biogenesis, selectively eliminated mitochondria can be replaced in a timely fashion by new mitochondria. Mitochondria possess unique DNA and proteins, and mitochondrial biogenesis mainly involves communication between the nucleus and the mitochondria. Peroxisome proliferator-activated receptor-γ coactivator-1α (PGC-1α) is an important nuclear transcription factor involved in mitochondrial biogenesis. PGC-1α can regulate the expression of nuclear respiratory factors 1 and 2 (NRF1 and NRF2), which are responsible for the regulation of genes involved in mitochondrial DNA (mtDNA) replication and the OXPHOS system [41, 42].

Mitochondrial biogenesis dysfunction plays an important role in the recovery phase of AKI. In a sepsis-induced AKI model, an initial decrease in PGC-1α expression in the acute phase and then an increase in parallel with recovering renal function have been observed, suggesting dysfunction in mitochondrial biogenesis. Similarly, PGC-1α knockout mice with persistent AKI also confirmed this theory [11]. These studies showed the restoration of renal function after targeting the PGC-1α pathway and further verified the existence of mitochondrial biogenesis dysfunction during AKI [43, 44].

#### Mitochondrial dynamics

In addition to the process of mitochondrial biogenesis mentioned above, mitochondrial dynamics constitute another important method of maintaining mitochondrial homeostasis. Mitochondria are highly dynamic organelles that constantly switch between fusion and fission under different physiological conditions. Mitochondrial dynamics are regulated by fusion proteins, including mitofusins 1 and 2 (Mfn1 and Mfn2), optical atrophy (OPA1) and fission proteins, such as dynamin related protein 1 (DRP1) [45, 46]. Normally, these proteins cooperate with each other to optimally balance between fission and fusion, ensuring basic physiological functioning of the mitochondria. During AKI, DRP1 is upregulated, and Mfn2 is downregulated, resulting in a mitochondrial tendency towards fission rather than fusion [46]. The role of DRP1 in the disruption of mitochondrial dynamics was first reported by Brooks et al. In a rat model of either I/R injury or cisplatin treatment, the inhibition of DRP1 was found to attenuate mitochondrial fragmentation [12]. In addition to DRP1, Mfn2 deficiency is also regarded as a risk factor for AKI due to its high sensitivity to Bax accumulation-mediated mitochondrial fragmentation under stressful conditions [47]. The latest finding pertaining to mitochondrial dynamics in the development of AKI is the role of OPA1. While fission proteins such as DRP1 are responsible for the cleavage of the mitochondrial outer membrane during AKI, OPA1, a key inner membrane fusion protein, has a substantial impact on inner membrane cleavage. In rat kidney proximal tubular cells cultured in an ATP-depletion injury environment, suppressed OPA1 proteolysis was reported to be associated with less mitochondrial fragmentation, Cyt C release and cell apoptosis. An in vivo study showed parallel results, confirming the protective role of the inactivation of OPA1 proteolysis in renal I/R injury [48].

Taken together, mitochondrial fragmentation due to enhanced mitochondrial fission during cell stress could result in accelerated Bax insertion and oligomerization in mitochondria, causing Cyt C leakage and finally renal tubular cell injury [49].

#### Mitophagy

In addition to mitochondrial dynamics, recent work has suggested the involvement of mitophagy in the pathophysiological process of AKI. The homeostasis of mitochondria relies on a series of quality control steps, the most important of which is mitophagy. Mitophagy is a selective form of autophagy in which autophagosomes engulf a single damaged mitochondrion or a cluster of damaged mitochondria. The autophagosome then subsequently fuses with a lysosome, which leads to the complete removal of the damaged organelles and proteins [50]. Dysregulation of autophagy is very common in the pathophysiological processes of cardiomyopathy, Crohn’s disease and neurodegenerative disorders [8]. The mitophagy pathway may be rapidly upregulated by hypoxia, oxidant injury and nutrient deprivation, all of which often occur during AKI. Therefore, it is not surprising that dysfunction of mitophagy may play a role in the development of AKI. Recently, various studies have confirmed that autophagy is rapidly activated in multiple models of AKI, and some studies have demonstrated this phenomenon occurring even prior to the clinical manifestations of tissue injury [51, 52]. For example, chloroquine blocking revealed that autophagy exacerbated cisplatin-induced tubular damage and renal function [53]. At genetic level, Atg5-knockout mice accumulated deformed mitochondria, indicating the impaired function of mitophagy. These mice were also susceptible to I/R injury [54]. In contrast, activation of the mitophagic pathway using rapamycin treatment might attenuate tubular damage [55]. The role of mitophagy during AKI is generally regarded as a renoprotective one that removes damaged mitochondria and restricts the release of pro-apoptotic substances. However, insufficient autophagy in the acute phase or excessive activation of this selective autophagic process in the recovery phase might also delay renal function recovery.

### MSC therapy targets mitochondrial dysfunction in AKI

**Fig. 2:**
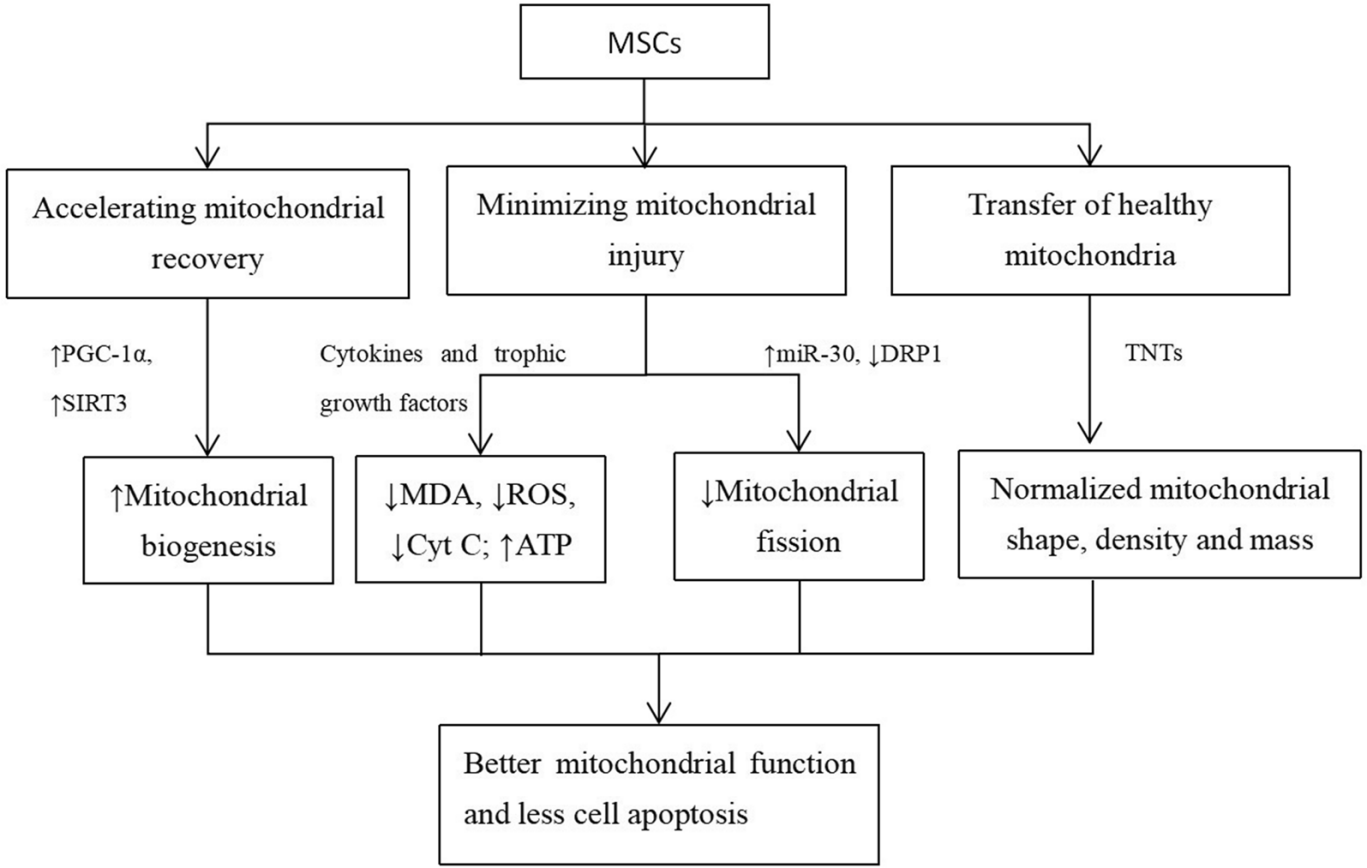
The mechanism by which MSC therapy targets mitochondrial dysfunction in AKI. MSCs are able to accelerate mitochondrial recovery, minimize mitochondrial injury and transfer healthy mitochondria to injured cells. These actions result in decreased levels of MDA, ROS and Cyt C, accompanied by reduced mitochondrial fission and enhanced mitochondrial biogenesis, finally inducing improved mitochondrial function and a reduction in cell apoptosis. *MSCs* mesenchymal stem cells, *PGC*-*1α* peroxisome proliferator-activated receptor-γ coactivator-1α, *SIRT3* sirtuin 3, *DRP1* dynamin related protein 1, *TNTs* tunneling nanotubes, *MDA* malondialdehyde, *ROS* reactive oxygen species, *Cyt C* cytochrome C, *ATP* adenosine triphosphate

Because mitochondrial dysfunction is a common phenomenon in the development of AKI, many approaches to improving mitochondrial function have been explored and have presented certain levels of therapeutic effects. These approaches are designed to achieve at least one of the following three goals: 1) minimizing mitochondrial injury; 2) accelerating mitochondrial recovery; and 3) direct transferring of healthy mitochondria (Fig. 2). As mentioned above, multiple drugs such as mito-Q, SS-31, midiv-1, etc. have been indicated to improve the renal outcome in diverse preclinical AKI models [56]. SS-31 has also been validated in a clinical trial [57]. However, all these drugs are prophylactic. This limitation may largely restrict their application in clinical situations because in most cases, the development of AKI is unpredictable. However, MSC-based therapy can still result in beneficial effects even after a kidney insult has already occurred. This could be a substantial advantage of MSC-based therapy over other mitoprotective interventions. Moreover, while pharmacologic interventions often target only one single aspect of injury, MSC-based therapy may have the advantage of acting through multiple mechanisms to promote mitochondrial functional recovery. Below, we will discuss the mechanism by which MSC therapy targets mitochondrial dysfunction in AKI. The associated articles are summarized in Table 1.

**Table 1:** Associated articles demonstrating the mechanisms by which MSC therapy target mitochondrial dysfunction in AKI

Year	Animal	AKI model	MSCs source	Outcomes	References
2017	Mice	Glycerol	Bone marrow	↑ATP, ↑Activation of PI3K/Akt pathway; ↓ROS, ↓Mitochondrial-apoptosis related proteins, ↓Cell apoptosis	Geng et al. [64]
2013	Rats	Cisplatin	Umbilical cord	↓Activation of mitochondrial apoptosis signaling, ↓MDA	Peng et al. [65]
2017	Rats	Cisplatin	Bone marrow	↑PGC-1α, ↑Activation of wnt/β-catenin pathway; ↓ROS	Jiao et al. [66]
2016	Rats	I/R	Wharton Jelly	↑miR-30; ↓Mitochondrial fssion, ↓Cell apoptosis	Gu et al. [68]
2017	Mice	Cisplatin	Umbilical cord	↑ATP, ↑PGC-1α, ↑NAD+, ↑SIRT3; ↑Mitochondrial exchange ↓ROS; Normalized mitochondrial shape, density and mass	Perico et al. [70]

*MSCs* mesenchymal stem cells, *AKI* acute kidney injury, *I/R* ischemia/reperfusion, *ATP* adenosine triphosphate, *ROS* reactive oxygen species, *MDA* malondialdehyde, *PGC*-*1α* peroxisome proliferator-activated receptor-γ coactivator-1α, *SIRT3* sirtuin 3

#### Minimizing mitochondrial injury

The release of Cyt C from damaged mitochondria no doubt plays a central role in mediating caspase activation during apoptosis [58]. The common view is that the influence of Bcl-2 family members on apoptosis is also based on their ability to regulate the permeability of mitochondrial membranes to ions and proteins [59]. The Bcl-2 family includes anti-apoptotic proteins such as Bcl-xl and Bcl-2 and pro-apoptotic proteins such as Bax, Bak, and Bad. [60]. The activation of Bax or Bak can induce Cyt C release, while the inhibition of Cyt C release by Bcl-2 and Bcl-xl is also well recognized. MSCs are able to secrete a series of cytokines and trophic growth factors, including hepatocyte growth factor (HGF) [61], vascular endothelial growth factor (vEGF) [62], and insulin-like growth factor-1 (IGF-1) [63], which may upregulate anti-apoptotic proteins, downregulate pro-apoptotic proteins, reduce Cyt C release and finally minimize mitochondrial injury during AKI. In a glycerol-induced AKI model, treatment with MSCs increased ATP production, decreased ROS levels, decreased the expression of mitochondrial apoptosis-related proteins and reduced tubular epithelial cell apoptosis, revealing the beneficial effects of MSCs in attenuating mitochondrial respiratory function and relieving renal injury [64]. Peng et al. transplanted MSCs into cisplatin-induced AKI rats. They found that the mechanism underlying the renal protective effects of infused MSCs might come from the reduced release of Cyt C from the mitochondria into the cytoplasm and the reduced level of malondialdehyde (MDA), indicating that MSCs can protect renal cells from mitochondrial-related apoptosis and oxidative damage [65]. Similarly, after injection with bone marrow MSCs-derived conditioned medium, cisplatin-induced AKI rats had reduced mitochondrial ROS levels and improved tubular cell morphology. The authors also confirmed that the beneficial effects were mediated by the activation of the wnt4/β-catenin pathway [66].

As mentioned above, mitochondrial fission is an important pathophysiological change during AKI and results in the leakage of apoptosis-related substances. Thus, the inhibition of mitochondrial fission could be another therapeutic target in AKI. Previous studies have demonstrated the beneficial role of mdivi-1, which is a pharmacologic DRP1 inhibitor, in blocking mitochondrial fragmentation and protecting kidneys against AKI [12, 43]. In addition to mdivi-1, miR-30 is also involved in the process of mitochondrial homeostasis by blocking DRP1 activation. Alleviation of mitochondrial fission through the miR-30/DRP1 pathway has been observed in myocardial tissues [67]. Based on this fact, Gu et al. conducted a series of studies to test the role of MSCs in the regulation of mitochondrial fission via the miR-30/DRP1 pathway. They applied extracellular vesicles derived from human Wharton Jelly MSCs (hWJMSCs) in a rat model of unilateral I/R AKI. Enhanced expression of miR-30, deactivation of DRP1, alleviation of mitochondrial fragmentation and reduction of cell apoptosis were observed, suggesting that MSCs might be involved in the modulation of mitochondrial fission via miR-30, thereby protecting the kidney from injury [68].

#### Accelerating mitochondrial recovery

In addition to minimizing mitochondrial injury, accelerating mitochondrial recovery is another useful strategy for restricting mitochondrial dysfunction during AKI. As mentioned above, PGC-1α may become a novel therapeutic target because of its important role in mitochondrial biogenesis. Previous studies have demonstrated that upregulating the PGC-1α pathway can stimulate mitochondrial biogenesis and accelerate mitochondrial and tubular function recovery [43, 69]. By promoting mitochondrial biogenesis, MSCs can exert similar effects, accelerating the recovery of normal mitochondrial mass and function. Perico et al. transplanted human umbilical cord-derived MSCs (UC-MSCs) into a cisplatin-induced AKI mouse model. Using electron microscopy, they found that compared with saline, treatment with UC-MSCs normalized the mitochondrial shape, density and mass in renal tissues [70]. To further explore the specific molecular mechanism underlying the protective effect of MSC therapy, the authors assessed the level of sirtuin 3 (SIRT3) during treatment. SIRT3 is an NAD+-dependent deacetylase that mainly participates in the management of mitochondrial oxidative stress [71]. More recently, there were studies confirming that the beneficial role of SIRT3 during AKI was associated with the recovery of mitochondrial dynamics [72, 73]. After conducting a series of experiments, the authors confirmed that the improved mitochondrial function might be due to the replenishment of NAD+, activation of SIRT3 deacetylase and upregulated transcription of PGC-1α following MSC treatment [70]. Similar results were found in another cisplatin-induced AKI rat model, in which PGC-1α expression and mitochondrial function were suppressed by cisplatin, an effect that was then reversed by MSC treatment [66].

#### Direct transfer of healthy mitochondria

Direct transfer of healthy mitochondria to injured cells is a more economical and faster option to improve mitochondrial function during acute injury compared to the two strategies mentioned above. MSCs are promising candidates as mitochondria donors due to their low immunogenicity and easy availability. Intercellular mitochondrial transfer from MSCs to target cells relies on the formation of tunneling nanotubes (TNTs), microvesicles, gap junctions and cell fusion [74]. Multiple studies have observed the phenomena of mitochondrial transfer from MSCs to injured cells during the healing process after acute respiratory distress syndrome [18], cardiomyopathy [75], damage to corneal epithelial cells [76] and infectious diseases [77]. Whether MSCs can similarly transfer mitochondria to renal tubular cells during AKI is unknown.

Mitochondrial transfer from MSCs to renal tubular cells has been documented in an in vitro cocultivation of rat renal tubular cells and human bone marrow-derived MSCs (BM-MSCs). After 3 h of coculturing, mitochondrial transport was observed in two directions between MSCs and renal tubular cells with the help of TNTs [78]. However, there is still some doubt about the role of mitochondrial transfer in AKI models because transplanted MSCs are only detected in peritubular areas, which are distant from the injured renal tubular cells. Perico et al. conducted a series of experiments to address this question. First, using the transfection method, they labeled mitochondria of two distinct populations of renal proximal tubular epithelial cells (RPTECs) with red or green fluorescent protein. After mixing and culturing in equal proportions, 18% of cells showed both red and green mitochondria, indicating mitochondrial transfer among them. Exposure to cisplatin reduced the percentage to 4.1%, which was then recovered to 25% by treatment with MSCs, suggesting that MSCs have a therapeutic effect on mitochondrial transfer. They also observed the formation of tubulin-rich protrusions, which are responsible for mitochondrial intercellular transport between cells through the activation of SIRT3. In vivo studies further confirmed the existence of complex interconnected microtubule networks in mice with cisplatin-induced AKI. Based on these results, the authors concluded that treatment with MSCs can enhance mitochondrial transfer and relieve cisplatin-induced renal injury, although the transferred mitochondria originated in adjacent relatively healthy renal tubular cells rather than MSCs [70].

So far, we have discussed the mechanism underlying the MSCs-mediated mitochondrial protective effects in AKI. However, the route by which MSCs are delivered is also an important factor. In the following section, we will discuss the impact of different MSCs delivery routes on their homing and engraftment into damaged kidneys.

### Optimal route for MSCs delivery in AKI

The optimal route for MSCs delivery is an important question that should be discussed. The prerequisite for the success of MSC therapy in AKI is the delivery of these cells to the damaged kidneys. Currently, the following four main delivery methods exist: intravenous, intra-arterial, intraperitoneal and intrarenal. To assess the impact of different routes on the homing and engraftment of MSCs, Wang et al. conducted a systematic review and concluded that intra-arterial delivery of MSCs induced a greater creatine reduction when compared with the intrarenal route or intravenous injection [79]. However, a subsequent meta-analysis in 2016 indicated that regardless of the delivery route, conditioned medium/extracellular vesicles derived from MSCs were not associated with improved renal function [80]. These inconsistent findings may confuse physicians when making clinical decisions. However, with the help of newly developed cell tracking techniques, it seems that the conflicting results originate from the same problem. Cell tracking techniques include positron emission tomography/single photon emission computed tomography, superparamagnetic iron oxide magnetic resonance imaging, ultrasound photoacoustic, fluorescence microscopy quantum dot and bioluminescent imaging [81]. After tracking the fate of transplanted MSCs in vivo, Du et al. found rare dyeing of MSCs in periglomerular or peritubular areas after intravenous injection, while most MSCs were retained in the lung capillaries [82]. Intrarenal injection also led to the localization of the MSCs near the injection site [83]. On the other hand, the rich blood flow and blood velocity in the artery facilitates more efficient homing and engraftment of MSCs, as mentioned by Semedo [84]. The small size of conditioned medium/extracellular vesicles derived from MSCs make it possible for them to freely pass through the lung capillaries and migrate toward the damaged kidney after intravenous injection [85]. These studies provided an explanation for the above-mentioned conflicting meta-analysis results and provide guidance for physicians choosing a route for the transplantation of different compositions of MSCs.

## Conclusions and future perspectives

In our review, we summarized mitochondrial dysfunction during AKI and the role of MSC therapy in targeting mitochondrial dysfunction in AKI. MSCs are able to minimize mitochondrial injury, accelerate mitochondrial recovery and induce the transfer of healthy mitochondria through a series of paracrine/endocrine actions, which make MSCs a promising candidate for AKI management.

Pharmacologic agents targeting mitochondrial function during AKI have shown promising results in animal models. Some of them have been tested in early clinical studies (NCT02397213, NCT01720030). Until now, despite the encouraging results in preclinical trials of using MSCs for AKI management, relevant clinical trials have shown conflicting results (NCT00733876, NCT01602328). A phase I clinical trial verified the safety and efficacy of MSCs in patients who were at high risk of AKI after cardiac surgery. In contrast, a sequential phase II, randomized, double-blind, multicenter trial revealed a negative result in the treatment of patients with post-cardiac surgery AKI. It seems that there still exists a large gap between scientific observation and clinical application.

Some concerns about the clinical application of MSCs in AKI patients should also be taken into consideration. First, due to the high degree of heterogeneity and multiple mechanisms of action of MSCs, the precise definition and determination of the potency of MSCs are urgently needed. Second, whether autologous or allogeneic MSC therapy is the better regimen for AKI treatment is still a matter of debate, considering their antigenicity. The third concern is the optimal dose of MSCs for clinical therapy in AKI due to the substantial variability in different studies. Finally, the potential risk of tumorigenicity should always be kept in mind by physicians.

We look forward to an optimistic future of MSC therapy in AKI. Understanding the pathophysiological changes during AKI and clarifying the detailed renoprotective mechanism of MSCs may further expand the success of MSC treatment in AKI patients.

## Abbreviations

AKI: acute kidney injury
MSCs: mesenchymal stem cells
I/R: ischemia/reperfusion
ATP: adenosine triphosphate
OXPHOS: oxidative phosphorylation
ROS: reactive oxygen species
MPT: mitochondrial permeability transition
Cyt C: cytochrome C
SS: Szeto-Schiller
PGC-1α: peroxisome proliferator-activated receptor-γ coactivator-1α
NRF1 and NRF2: nuclear respiratory factors 1 and 2
mtDNA: mitochondrial DNA
Mfn1 and Mfn2: mitofusins 1 and 2
OPA1: optical atrophy
DRP1: dynamin related protein 1
HGF: hepatocyte growth factor
vEGF: vascular endothelial growth factor
IGF-1: insulin-like growth factor-1
MDA: malondialdehyde
hWJMSCs: human Wharton Jelly MSCs
UC-MSCs: umbilical cord derived MSCs
SIRT3: sirtuin 3
TNTs: tunneling nanotubes
BM-MSCs: bone marrow derived MSCs
RPTECs: renal proximal tubular epithelial cells
